# Construction and validation of competency frameworks for the training of nurses in emergencies*

**DOI:** 10.1590/1518-8345.2631-3061

**Published:** 2018-10-25

**Authors:** Fernanda Berchelli Girão Miranda, Alessandra Mazzo, Gerson Alves

**Affiliations:** 1 Universidade de São Paulo, Escola de Enfermagem de Ribeirão Preto, PAHO/WHO Collaborating Centre for Nursing Research Development, Ribeirão Preto, SP, Brazil.; 2 Universidade de São Paulo, Faculdade de Medicina de Ribeirão Preto, Ribeirão Preto, SP, Brazil.

**Keywords:** Nurses, Clinical Competence, Education, Education, Nursing, Nursing Assessment, Emergencies

## Abstract

**Objective:**

to build and validate competency frameworks to be developed in the training of nurses for the care of adult patients in situations of emergency with a focus on airway, breathing and circulation approach.

**Method:**

this is a descriptive and methodological study that took place in three phases: the first phase consisted in a literature review and a workshop involving seven experts for the creation of the competency frameworks; in the second phase, 15 experts selected through the Snowball Technique and Delphi Technique participated in the face and content validation, with analysis of the content of the suggestions and calculation of the Content Validation Index to assess the agreement on the representativeness of each item; in the third phase, 13 experts participated in the final agreement of the presented material.

**Results:**

the majority of the experts were nurses, with graduation and professional experience in the theme of the study. Competency frameworks were developed and validated for the training of nurses in the airway, breathing and circulation approach.

**Conclusion:**

the study made it possible to build and validate competency frameworks. We highlight its originality and potentialities to guide teachers and researchers in an efficient and objective way in the practical development of skills involved in the subject approached.

## Introduction

There are numerous challenges in the training of health professionals in the educational institutions of the different professional categories when it comes to promoting the training of students, preparing them to society to become qualified professionals who meet not only the expectations of the health system, but mainly the real needs of the population.

One of the main aspects that may hamper the achievement of this goal is possibly the difficulty to make the necessary changes in the curricular matrices of the health courses within the institutions to overcome traditional education and incorporate teaching and learning methodologies based on competencies, which have their major advances described in the teaching of American medicine^1^ ^-^ ^5^. Studies on the development of competency frameworks such as the CanMEDS Framework^6^ ^-^ ^7^, the Milestones^8^ ^-^ ^10^, the Tomorrows Doctors in the UK and the Scottish Doctor in Scotland started in the early 1990s^11^.

Competency frameworks are descriptions of the knowledge, skills, and attitudes pertaining to each competency expected during the students’ training. They are organized in a framework that shows the results of the progressive development of students based on competencies, since their insertion in the university until post-graduate training^12^ ^-^ ^13^.

In a narrative way, they describe the competencies that must be repeatedly demonstrated during curricular schedules in clinical environments with different levels of complexity^14^, allowing the possibility of a feedback to stimulate changes in observed behaviors, as well as a greater precision in the application of evaluative scales^15^ ^-^ ^17^. Competency frameworks have been expanded in varied medical specialties such as General Surgery^12^ ^,^ ^18^ ^-^ ^19^, Pediatrics^20^ ^-^ ^22^, Urology^23^, and specially Emergency^13^ ^,^ ^17^ ^,^ ^23^ ^-^ ^27^, in which worldwide advances in multiprofessional care occur^28^. In nursing training, however, competency frameworks represent a topic yet to be explored, because the content taught often depends on the teachers’ conceptions and does not have a specific discipline to be approached^29^.

The situation is even more worrying when associated with the shortage of active learning methodologies and of opportunities for the practical experience of future professionals. Pedagogical strategies are essential to promote integrative construction of knowledge, reflexive observation and the closest possible approximation with the real environment, so as to promote the confidence of the students^30^.

There is a lack of studies published in scientific journals specifically on professional competence of nurses in emergencies. According to the study^31^, the theme is often exclusively directed to the management area.

In this context, it is essential to review the nursing education and ensure that these professionals have an effective participation in patient care during emergency situations. Patients with a compromised airway quickly become unstable and run the risk of cardiorespiratory arrest^32^. Recent studies^33^ ^-^ ^34^ emphasize the need to evaluate strategies for ventilatory support, because nurses need to have the knowledge, ability and competent clinical reasoning to anticipate, monitor and intervene when any complication arise from ventilatory support^35^. Critical hemodynamic events usually occur along with predictable progressive deterioration, with signs and symptoms anticipated by physiological changes^36^.

In situations of emergency, airway, breathing and circulation (ABC) approach must take place systematically and rapidly, with identification of possible disorders and onset of immediate treatment. This is applicable in all traumatic and non-traumatic emergencies, either in extra-hospital environments where there is usually no life support equipment for rescue maneuvers, or in more advanced settings such as pre-hospital mobile service, emergency rooms, general hospital wards, or in intensive care units.

The uniform adoption of the ABC approach among team members is capable of improving joint performance. The training of team members can have an impact on the outcomes, the recognition of professionals and the management of care for patients in acute or critical situations, allowing increased self-confidence and lower reluctance on the part of professionals^37^.

This study aims to build and validate competency frameworks to be developed in the training of nurses to provide care for adult patients in urgent situations with a focus on the ABC approach.

## Method

Descriptive, methodological study of construction and validation of competency frameworks to be developed in the training of nurses regarding care for adult patients during ABC approach in situations of traumatic and non-traumatic urgency.

The study was developed in three phases: the first phase consisted in a literature review on the theme, with the search, synthesis and analysis of studies. Then, a workshop was held at a public university in the countryside of São Paulo, with seven experts to build the competency frameworks. The recruitment of experts was done by means of the “snowball technique”, in which a sample is created by means of indications of people who have in common characteristics that are of interest for the research^38^ ^-^ ^39^. Following this technique, one professional (key informant) indicated the name and the electronic address of six professionals that met the inclusion criteria of the study, to whom invitations were sent. The inclusion criteria for selection of experts were adapted from Fehring’s^40^ reference as follows: health professionals with at least one year experience in adult patient care in situations of emergency, in the context of care and/or teaching.

During the workshop, the seven experts participating in the study were trained for familiarization with the theme and they were requested to build the competency frameworks for the training of nurses in the adult patient care in traumatic and non-traumatic emergencies, using the ABC approach.

The experts were randomly distributed into three different groups, where each group was responsible for building the specific competency frameworks for each item of the ABC algorithm. They also filled a form for biographical and professional characterization and signed the Informed Consent Form (ICF).

In the second phase of the study, the competency frameworks prepared in the first phase were validated. The “snowball” technique was also used in this phase^38^ ^-^ ^39^. On expert (key informant) was requested to indicated the name and e-mail address of three professionals who met the inclusion criteria of the study. Every new participant was invited to do the same, i.e., indicate peers who could contribute to the study.

The Fehring^40^ criteria were adapted and used to select experts to be included in the study: being a health professional; being involved in care and/or teaching with at least one certificate of clinical practice (specialization) in the area of interest of the study; having master’s degree with thesis in the area of interest of the study; having PhD degree with dissertation in the area of interest of the study; having clinical experience of at least one year; having published relevant research in the area of interest and having published articles on the theme in reference journals. To be considered an expert, the participant had to meet at least one of the items above mentioned.

Seventy-six professionals were indicated through the “snowball” technique^38^ ^-^ ^39^ and met the criteria adapted from Fehring^40^.

The invitation to participate in the research was sent to all the 76 experts via e-mail with a web access link, which directed the person to the electronic form, made available through Google Docs Off line^®^. Upon clicking the link, the Informed Consent Form (ICF) was promptly opened for completion, being this step a mandatory condition for the opening of the following pages with the biographical and professional characterization forms, editing manual, and the competency frameworks for face and content validation.

The invited experts were requested to return the data collection instruments within a maximum period of 30 days. Fifteen (15) professionals responded to the validation of the material built. In this phase of face and content validation, we used the Delphi Technique, a method that represents a methodological research strategy whose objective is to obtain a maximum of agreement in a group of experts on a certain theme^41^.

The third phase of this study occurred after the analysis of content of the considerations and suggestions provided by the experts for the material to be validated. Content analysis focuses on a set of techniques to perform analyses during the process of reading the results of the contributions of experts, systematized through essential concepts, described by: objectivity, systematicity, manifest content, registration units, context units, creation of categories, analysis of categories, inference, and conditions of production^39^.

For content analysis^39^, the data in the answers were categorized, classified and quantified for interpretation of the results. After an exhaustive reading of the primary categorization of the data, these were realigned to each competency framework related to each ABC framework and according to the topics expressed in the original messages provided by the experts. Then, data were related in meaning units and contextual units, which cover the messages in full length according to the participants.

After data organization, the main components were identified to compose the competency frameworks to be promoted in the training of nurses for adult patient care involving the ABC approach in situations of traumatic and non-traumatic urgency.

The Content Validation Index (CVI) was then calculated to assess the agreement among experts on the representativeness of each item in the tables. For this study, the minimum index of 0.80 for each item in the table was considered acceptable for calculating the CVI^42^.

After analysis, a new version of the competency frameworks was created and, after that, a second round of opinions was requested^43^. The 15 participating experts received a new e-mail with the redesigned competency frameworks and were requested to return the material within a maximum time of 30 days. The participants of this phase were 13 experts, who characterized the agreement on the material presented. Data were collected from experts was from March to August 2017, through ethical authorization under Opinion 55082716.5.0000.5393.

## Results

Table 1 describes the characterization of the participating experts in each validation phase of the competency frameworks.

**Table 1:** Characterization of the participating experts in each validation phase of the competency frameworks. Ribeirão Preto, SP, Brazil, 2017

Variables	Characterization of experts sharing in the first phase f (%)	Characterization of experts sharing in the second phase f (%)	Characterization of experts sharing in third phase f (%)
Number of participants	7 (100%)	15 (100%)	13 (100%)
Sex			
Male	2 (28.6%)	6 (40.0%)	5 (38.4%)
Female	5 (71.4%)	9 (60.0%)	8 (53.3%)
Professional qualification			
Nursing	6 (85.7%)	15 (100%)	13 (100%)
Medicine	1 (14.3%)	0	0
Postgraduate training*			
Specialization	4 (57%)	10 (66.7%)	10 (76.9%)
Master degree	5 (71.4%)	15 (100%)	13 (100%)
PhD degree	3 (42.8%)	7 (46.7%)	7 (53.8%)
Postdoc	1 (14.3%)	1 (6.7%)	1 (7.69%)
Current professional area of activity			
Care	1 (14.3%)	4 (26.7%)	4 (30.7%)
Teaching	5 (71.4%)	6 (40.0%)	4 (30.7%)
Care and teaching	1 (14.3%)	5 (33.3%)	5 (38.4%)
Research publications and/or articles on the subject	6 (85.7%)	6 (40.0%)	6 (46.1%)

* The experts reported more than one academic title

In the first phase of the study, the experts participating in the workshop built the ABC competency frameworks to evaluate frameworks related to the progression of students, which were classified into three distinct levels: level 1 (student before immersion in the supervised curricular internship), level 2 (student immersed in the supervised curricular internship) and level 3 (nurse).

This classification of progression of students was established after a consensus of the experts based on Resolution CNE/CES N^o^ 3 of November 7, 2001, which establishes the National Curricular Guidelines of the Nursing Undergraduate Course^1^, which states in Art. 7: *“In Nursing training, in addition to the theoretical and practical contents developed during training, the courses are required to include in the curriculum a supervised internship in general and specialized hospitals, outpatient clinics, basic health service network, and communities in the last two semesters of the Undergraduate Nursing Course”.*

Thus, as all Higher Education Institutions offering Nursing courses have adopted the Curriculum Guidelines^*^, the supervised curricular internship is mandatory in the last two semesters of training, characterizing it as the fundamental framework in the students’ progression and development.

In the following phases, the experts contributed to the validation using the Delphi technique^41^. The suggestions received from the experts had their content analyzed, and it was concluded that there was a consensus on the content presented, resulting in the tables of competency frameworks presented below.

Agreement among experts on the representativeness of the items in relation to the content of the tables was presented through the CVI. In the third phase, some items from the first analysis presented a CVI below 0.80; thus, the comments and suggestions provided by the experts were considered for the possibility of adjustments, and then the material was returned to the participants, resulting in CVI values ≥ 85% for all items in the final analysis.

The final result represents the consensus among experts on the competency frameworks involving knowledge, skills and attitudes considered to be minimally necessary for nurses to prevent instabilities, contribute to the treatment and recovery of adult patients during ABC approach in traumatic and not traumatic urgencies.

Each cell in the table reveals a competency framework to be demonstrated by the students within their level of progression. Competency frameworks are present in greater number mainly at the initial levels of training, what is expected in relation to the students’ developmental needs. Thus, the level 3 (nurse) has consequently a higher level of empty cells.

**Figure 1:**
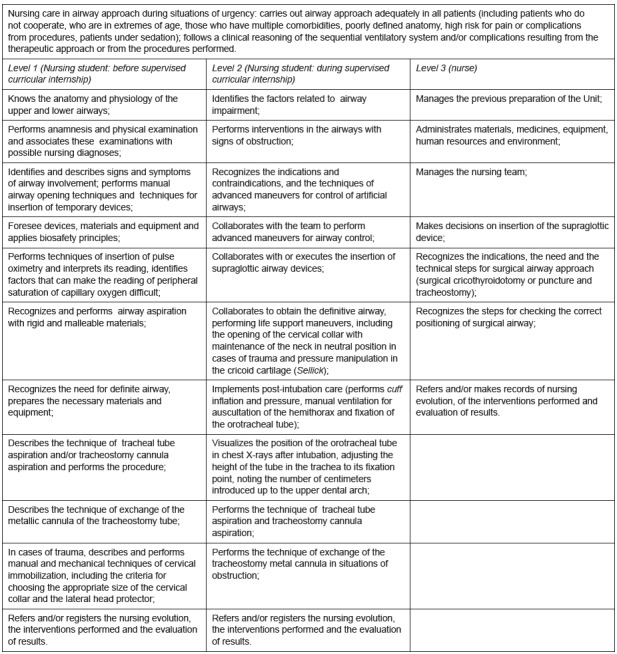
Competency frameworks prepared and validated for nursing care in airway approach in situations of urgency. Ribeirão Preto, SP, Brazil, 2017

Figure 1 below presents the competency frameworks prepared and validated for nursing care in airway approach in situations of urgency.

**Figure 2:**
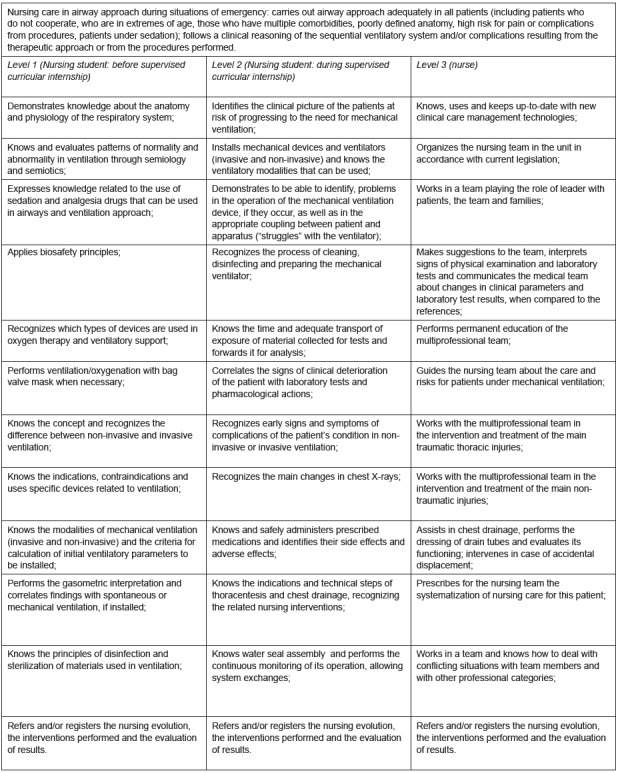
Competency frameworks prepared and validated for nursing care in airway approach in situations of urgency. Ribeirão Preto, SP, Brazil, 2017

Figure 2 below presents the competency frameworks created and validated for nursing care in the airway approach in situations of urgency.

**Figure 3:**
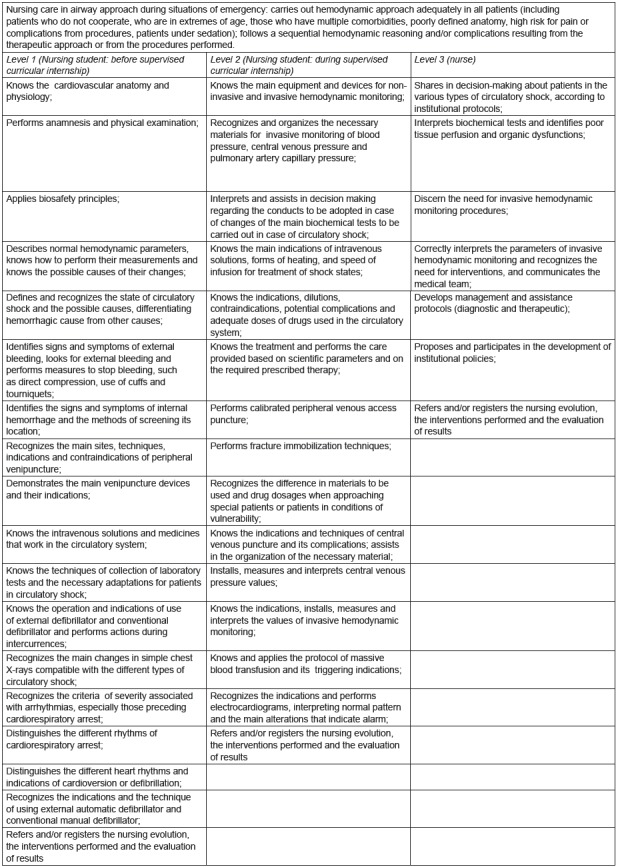
Competency frameworks built and validated for nursing care in the approach of hemodynamic state in situations of urgency. Ribeirão Preto, SP, Brazil, 2017

Figure 3 below presents the competency frameworks constructed and validated for nursing care in the hemodynamic state approach in emergency.

## Discussion

The competency frameworks to be developed in nursing training in adult patient care in situations of traumatic and non-traumatic urgency focusing on the ABC approach aims to support the training and work of nurses in the most varied of situations experienced and referred in the clinical practice.

With the Competency frameworks, the ability to assemble measures in different contexts makes it possible to identify trends in student performance, revealing the need for improvement. They guide teachers in the process of developing their programs within the curricular matrix throughout the course, as well as in the evaluation process, generating a model that can be shared among diverse schedules regarding the expected knowledge, skills and behaviors of students^15^.

The data provided by the competency frameworks serve as indicators of curriculum performance. The results of the competency frameworks used for a student group show whether the results of the desired competencies are being achieved and is clearly useful as a source of data for discussions on improvements and monitoring of the potential impacts of curricular changes^15^.

It is important to note, however, that in other validation studies some experts have pointed out that although the content of competency frameworks is important for the training of health professionals and provides a structure for demonstrating the progression of the students, competency frameworks need a differentiation for each specialty, requiring time and physical and financial investments from educational institutions^19^.

In terms of application, it is discussed the context in which the competency frameworks will be specified and observed, the number of frameworks grouped within each level, or the total number of competencies and sub-competencies to be developed and evaluated, which may be detrimental to practice for the evaluation, and may need adjustments on the part of the educational institution^19^ ^,^ ^23^. These questions led to the introduction of the concept of *Entrustable Professional Activities* (EPAs)^9^ ^,^ ^44^ ^-^ ^45^.

However, other studies present positive perceptions of trainers about this evaluation process when compared to systems previously used in some institutions, with emphasis on the benefits of the competency-based education strategy, students’ performance evaluation, and the ability to provide impartial feedback ^19^ ^,^ ^23^.

A great current questioning among professional education scholars is that if we really want to transform nursing education and practice, we must believe and find proposals to implement the positive results of educational strategies directed to the environment of practice^28^.

The discussion about the need for changes in the training of competent nurses has existed for more than three decades. However, the lack of standardization of essential competencies combined with a limited number of validated assessment tools has been a challenge^46^ which, in critical situations, can put both the patient and the professional at risk.

In the results found in this study, we saw that the proposed methodology made it possible to reach a consensus among experts in the assembling of essential skills for the training of nurses in the theme studied. The proposal is that these competency frameworks are influential in curricular training strategies, and some of these competency frameworks are probably taught in traditional curricula, but in disconnected and poorly integrated moments of training. The important point to note is that it must be considered that in order to develop each competency framework at each level of training progression, there is a previous and even simultaneous theoretical and practical context or even contextualized by the pedagogical plan of the course. Competency frameworks allow the students to visualize their current status and reflect on what behaviors are needed for their professional training^23^ ^,^ ^47^. Even more important than an evaluation process, competency frameworks allow the students to visualize a learning itinerary that must continue at each moment of their training^12^ ^-^ ^16^.

Study^48^ reinforces the need for proposals to objectively assess and record the competency of nurses in urgencies, stating that it is not appropriate to use a single method of competency development and evaluation. A recent Brazilian study^49^ presented a matrix of basic nursing and associated skills for to work in emergencies and highlighted the shortcomings in the national literature.

This study, although specifically related to the theme of urgency, may be a future reference for other nursing education areas, becoming a tool for teachers to reflect on the training processes, and the development and evaluation of curricula. Several authors regard competency frameworks as important to guide evaluators, resulting in a mental model that can be shared in all curricular programs as to the knowledge, skills and behaviors expected of students ^15^ ^,^ ^19^ ^,^ ^23^ ^,^ ^47^.

Regarding the limitations found in this study, it is important to point out that other investigations on the subject related to the development and evaluation of nurses were not identified, nor were they directed to the area of ​​urgency in the profession, which exalts its found, thus hindering the comparison of the results found here with other investigations. The methodological approach is characterized as a restriction of results, because the number of experts participating in the study was reduced in relation to the number of people invited. Another bias is related to the “snowball” technique, considering the fact that the people accessed by the method are the most visible in the participating population, which prevents the generalization of the results.

Given the potential presented, it is expected that this study contribute to the design of a matrix of development and evaluation of competencies in the theme proposed in the training of nurses. The study suggests the investment in further research to help in the creation of competency frameworks as proposed, not only in the area of ​​urgency, but also in other areas of nursing teaching and research.

## Conclusion

The study resulted in different competency frameworks to be developed in the training of nurses regarding adult patient care in traumatic and non-traumatic urgencies in the ABC approach, with the potential to guide teachers and researchers in an efficient and objective way for practical development in the theme.

We hope that the competency frameworks complement the nursing training process by mutually collaborating with students and teachers in an objective and effective way to develop and evaluate competencies, as competency-based educational advances are increasingly needed to meet the needs of the population, impacting patient care and safety.

## Footnotes

1 National Council of Education. (2001). Opinion CNE/CES n. 3, from November 7, 2001. Provides for the National Curricular Guidelines of the Undergraduate Nursing Course [Internet legislation]. Brasília. http://portal.mec.gov.br/cne/archives/pdf/CES03.pdf
